# Aortic valve replacement in a patient with MPO-ANCA-positive Goodpasture disease

**DOI:** 10.1186/s40792-016-0230-x

**Published:** 2016-09-21

**Authors:** Go Kataoka, Ryota Asano, Atsuhiko Sato, Wataru Tatsuishi, Kiyoharu Nakano

**Affiliations:** Department of Cardiovascular Surgery, Tokyo Women’s Medical University Medical Center East, 2-1-10, Nishiogu, Arakawa-ku, 116-8567 Tokyo, Japan

**Keywords:** Goodpasture disease, MPO-ANCA, Interstitial pneumonia

## Abstract

Goodpasture disease (GD) is a rare autoimmune disorder characterized by the development of pathologic autoantibodies against both glomerular and alveolar basal membranes. Approximately one third of the patients with GD are also positive for anti-neutrophil cytoplasmic antibody (ANCA). In this case report, a 74-year-old woman was diagnosed as having myeloperoxidase (MPO)-ANCA-positive GD with severe aortic valve stenosis (AS). She underwent immunosuppressive therapy and plasmapheresis that led to GD remission. Whether a cardiac surgery affects a MPO-ANCA-positive GD in remission is unknown. We reported the outcomes after aortic valve replacement for severe AS in a patient with MPO-ANCA-positive GD.

## Background

Anti-glomerular basement membrane (GBM) disease or Goodpasture disease (GD) is an autoantibody-mediated autoimmune small vessel vasculitis that generally presents as a pulmonary–renal syndrome. This rare disease has an annual incidence of 0.5–1 case per million and demonstrates a bimodal age distribution [1]. Up to one third of patients with anti-GBM disease are also positive for anti-neutrophil cytoplasmic antibody (ANCA), mainly with specificity to myeloperoxidase (MPO). While anti-GBM disease is generally considered a non-relapsing illness, ANCA-positive small vessel vasculitis has a relevant risk of relapse that demands maintenance therapy after induction of remission [2]. The factors that affect a MPO-ANCA-positive GD in remission period are unknown.

In this case report, we describe the outcomes after aortic valve replacement (AVR) for severe aortic valve stenosis (AS) in a 74-year-old woman with MPO-ANCA-positive GD.

## Case presentation

**Fig. 1:**
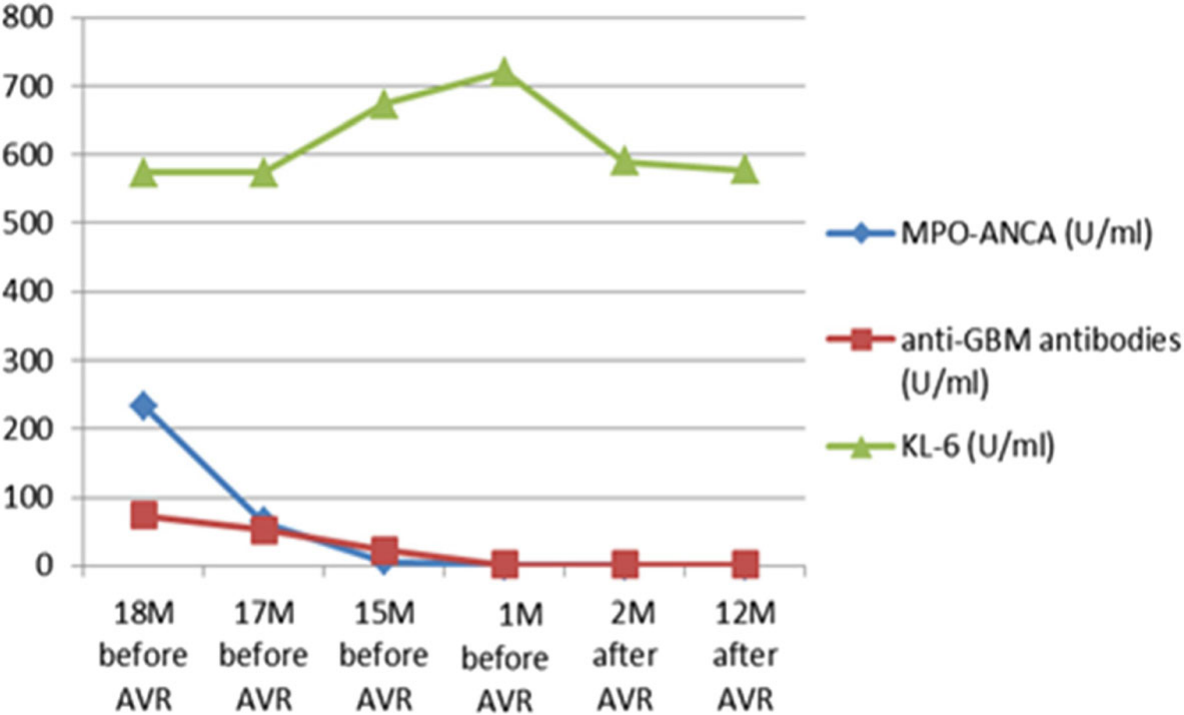
Preoperative and postoperative course in MPO-ANCA, anti-GBM-antibody, and KL-6. The patient received immunosuppressive therapy and plasmapheresis for MPO-ANCA-positive GD before 18 M prior to AVR. *MPO-ANCA* myeloperoxidase-anti-neutrophil cytoplasmic antibody, *anti-GBM-antibody* anti-glomerular basement membrane antibodies, *AVR* aortic valve replacement, *GD* Goodpasture disease, *M* months

A 74-year-old woman presented with cough, hemosputum, exacerbation of exertional dyspnea, appetite loss, and fatigue. She had a previous history of hypertension. On physical examination, inspiratory crackles in both lower lungs and a Levine III/VI systolic murmur on the second left sternal border were present. Blood tests showed markedly raised creatinine (1022 μmol/l), low hemoglobin (7.3 g/dl), low albumin (2.5 g/dl), high KL-6 (574 U/ml; normal <500 U/ml) (Fig. 1), positive anti-GBM antibody (73 U/ml; normal <3.0 U/ml), positive MPO-ANCA (233 U/ml; normal <3.5 U/ml) (Fig. 1), and high C-reactive protein (1.65 mg/dl; normal <0.21 mg/dl). She was admitted and received further examination.

Chest radiograph showed bilateral patchy pulmonary infiltrates in the middle and lower lung field. Atypical honeycomb lung was noted significantly in the subpleural portion and lower field of the lung on computed tomography (CT). CT finding showed usual interstitial pneumonia (IP) pattern. Transthoracic echocardiography demonstrated severe AS. Renal biopsy exhibited a necrotizing crescentic glomerulonephritis in the pathological examination. Acute renal failure was considered to be caused by rapidly progressive glomerulonephritis. She was diagnosed as having MPO-ANCA-positive GD with severe AS. Pulsed immunosuppressive therapy using methylprednisolone and plasmapheresis were performed immediately. These therapies improved her respiratory condition but did not improve her renal dysfunction. Maintenance dialysis was then introduced. Anti-GBM antibody and MPO-ANCA value dropped to 23 and 5.9 U/ml (Fig. 1), and the general condition of the patient improved. Therefore, she was discharged after about 2 months of hospitalization. Methylprednisolone pulses followed by prednisone was tapered up to 5 mg/day for a year, and MPO-ANCA-positive GD was considered to be in remission. She was introduced to the Department of Cardiovascular Surgery in our hospital for AS treatment.

**Fig. 2:**
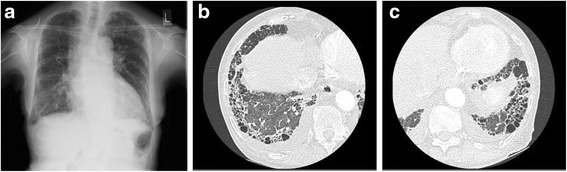
**a** Chest radiography 1 month before AVR. Chest radiography showed a cardiothoracic ratio of 63 % and bilateral infiltration shadow. **b** CT findings of the right lower fields of the lung. **c** CT findings of the left lower fields of the lung. CT indicated usual interstitial pneumonia pattern. *AVR* aortic valve replacement, *CT* computed tomography

Physical examination at the time of admission indicated a regular pulse of 82 beats/min, blood pressure of 130/80 mmHg, and a Levine III/VI systolic murmur on the second left sternal border. Chest radiography showed a cardiothoracic ratio of 63 % and bilateral infiltration shadow (Fig. 2a). His blood test results are as follows: creatinine, 302 μmol/l; hemoglobin, 9.9 g/dl; albumin, 3.8 g/dl; KL-6, 720 U/ml (Fig. 1); anti-GBM antibody, 0.9 U/ml; MPO-ANCA, 0.6 U/ml (Fig. 1); and C-reactive protein, 0.13 mg/dl.

The electrocardiogram showed sinus rhythm and ST depression with strain pattern in the I, aVL, and V5–V6 leads, which indicated left ventricular hypertrophy. Transthoracic echocardiography demonstrated an ejection fraction of 76 %. The left ventricular end-diastolic/end-systolic dimension, peak/mean pressure gradient through the aortic valve, and aortic valve area were 52/29 mm, 152/95 mmHg, and 0.76 cm^2^, respectively. Severe AS was recognized. Coronary angiography confirmed no significant stenosis in the coronary artery. Abnormal pressure value was absent in the right heart catheter test. The cardiac output and cardiac index were 6.4 and 4.5 l/min/m^2^, respectively. CT indicated usual IP pattern in the lung (Fig. 2b, c). The vital capacity (VC), %VC, forced VC, and FEV 1.0 % in the pulmonary function test were 2.07 l, 92 %, 2.08 l, and 86 %, respectively. Blood gas analysis (BGA) in the room air indicated a pH of 7.37, PO_2_ of 89.8 mmHg, and PCO_2_ of 42.9 mmHg. Lung function was maintained in the lower limit of normal.

After consultation with an anesthesiologist and a pulmonologist about her condition, we determined that cardiac surgery could be performed because GS and IP were considered inactive based on the data.

Surgery was performed through median sternotomy. Betamethasone (235 mg) was used upon initiation of cardiopulmonary bypass (CPB). CPB was established via an ascending aortic cannulation and right atrium drainage. A venting tube was placed in the left atrium through the right superior pulmonary vein. After cardiac arrest, aortotomy was performed. The aortic valve was tricuspid and severely calcified. A 21-mm Carpentier-Edwards PERIMOUNT (CEP) Magna Ease pericardial prosthesis (Edwards Lifesciences, Irvine, CA, USA) was implanted after valve excision. The patient was weaned off cardiopulmonary support uneventfully. Intraoperative administration of hydrocortisone sodium succinate (200 mg) and red cell concentrates (560 ml) was carried out. The CPB time and operative time were 75 and 148 min, respectively. She was extubated two and a half hours after the operation.

**Fig. 3:**
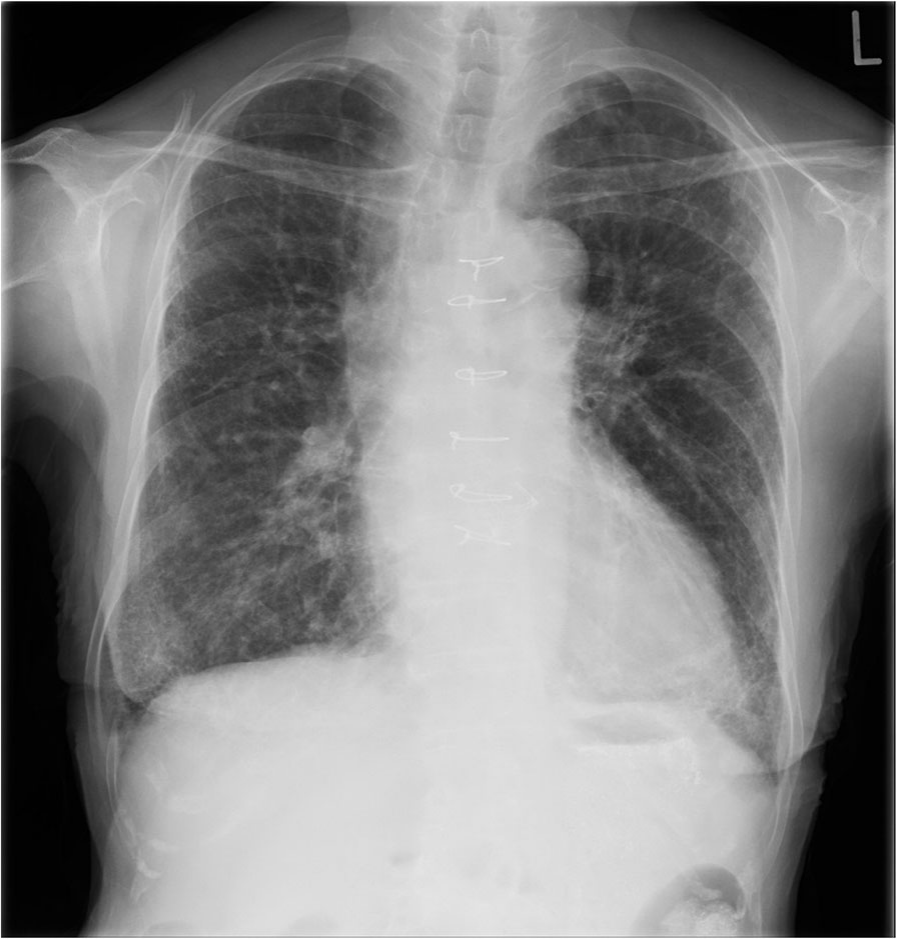
Chest radiography 12 months after AVR. Exacerbation findings were not confirmed in chest radiograph 12 months after AVR. *AVR* aortic valve replacement

She could be discharged at postoperative day 15 without GD relapse and IP exacerbation. Her condition was stable 1 year after the AVR. Her blood tests showed KL-6 of 578 U/m (Fig. 1), anti-GBM antibody of 1.0 U/ml, and MPO-ANCA of 2.0 U/ml (Fig. 1). Exacerbation findings were not confirmed in chest radiograph (Fig. 3). The patient continued to take 5 mg of prednisone daily.

### Discussion

Anti-GMB disease is a rare autoimmune disorder. The antigenic epitope within the non-collagenous-1 (α3 NCI) domain of type IV collagen in the GBM is well defined, and the confined expression of this collagen to glomerular and alveolar basement membrane leads to organ specificity of the disease. The deposition of anti-GBM antibody in glomerular and alveolar basement membranes induces glomerulonephritis and pulmonary hemorrhage [1, 3].

On the other hand, ANCA-associated systemic vasculitis, which is characterized by necrotizing inflammation of the small vessels, is closely related to ANCA directed to proteinase-3 or to MPO [4]. About 5 % of ANCA-positive patients have positive anti-GBM antibody, and 32 % of anti-GBM antibody-positive patients also have positive ANCAs. Furthermore, 82 % of patients with both antibodies have MPO-ANCA [5].

Double-positive (both anti-GBM antibody and ANCA antibody positive) patients have higher relapse rate than those with anti-GBM antibody alone [1]. This result is attributed to ANCA-positive small vessel vasculitis, which has a risk of relapse after induction of remission [2]. In this case, performing an AVR is considered difficult because of the possibility of MPO-ANCA-positive GD recurrence. Both genetic and environmental factors, such as hydrocarbon exposure, smoking, extracorporeal lithotripsy, virus, and *Clostridium botulinum*, are implicated in the development of anti-GBM disease [6]. However, factors that exacerbate GD are unknown. Fortunately, AVR used by CPB did not affect the patient with MPO-ANCA-positive GD in remission.

Etter et al. [7] reported that antibodies to human lysosomal membrane protein 2 (hLAMP2) could be an early indicator of double-positive GS relapse. Measuring anti-hLAMP2 antibody as well as anti-GBM antibody and ANCA antibody can be helpful in the long-term follow-up of patients.

Whether the IP was idiopathic or secondary due to double-positive GD in this case is unclear; thus, predicting how the IP is affected by the AVR is difficult. Prior to the operation, KL-6 was 720 U/ml, BGA was normal, and the lung function of the patient was maintained in the lower limit of normal. In consultation with the pulmonologist, we determined that surgery could be performed. Risk factors for acute exacerbation of IP are also unknown. The acute exacerbation of IP can cause death, particularly in those with honeycomb lung. Tanaka et al. [8] reported that performing endotracheal intubation without using a muscle relaxant is important. They also reported that postoperative care using a ventilator should also be performed while maintaining a low airway pressure and the PaO_2_ at ~80 Torr when total arch replacement is performed for aortic arch replacement with chronic IP.

Similarly, postoperative management by using a ventilator in this case maintained a low airway pressure and the PaO_2_ at ~80 Torr. In addition, intraoperative administration of 200 mg of hydrocortisone sodium succinate as steroid cover was performed. These attempts may help prevent exacerbation of GD and IP.

## Conclusions

This may be the first case demonstrating AVR with MPO-ANCA-positive GD. Based on the clinical findings, the GD and IP were in remission and inactive phase. Therefore, good early- and long-term results were obtained. However, the risk factors that exacerbate GS and IP remain unknown, and the possibility of GS relapse is present. Close follow-up is needed in the future.

## Consent

Written informed consent was obtained from the patient for publication of this case report and any accompanying images.
